# Comparison Between High-Flow Nasal Cannula (HFNC) Therapy and Noninvasive Ventilation (NIV) in Children With Bronchiolitis-Associated Acute Respiratory Failure: A Systematic Review and Meta-Analysis

**DOI:** 10.7759/cureus.114308

**Published:** 2026-08-10

**Authors:** Zeinh H Fardan, Assraf Abu Ayish, Rifal H AlQahtani, Noor M Naser, Noura Alali, Aliyah Aldabli, Atheer A Alghamdi, Sama Alghayyadh, Manar N Alahmri, Abrar K Bakhsh, Mohammed Alaslani, Zainab A Almarzooq, Mayar A Abduljawad, Halah Alasiri, Ayah M Banaja

**Affiliations:** 1 Child Health, King Khalid University, Abha, SAU; 2 Pediatric Intensive Care Unit, Barzilai Medical Center, Beersheva, PSE; 3 Medicine, King Khalid University, Abha, SAU; 4 Medicine, King Abdullah bin Abdulaziz University Hospital, Manama, BHR; 5 Medicine, Almaarefa University, Riyadh, SAU; 6 Pediatrics, Abha Maternity and Children Hospital, Abha, SAU; 7 Medicine and Surgery, Fakeeh College for Medical Sciences, Jeddah, SAU; 8 Medicine, Aljouf University, Aljouf, SAU; 9 Oral Medicine, East Carolina University School of Dental Medicine, Greenville, USA; 10 Pediatrics, Ministry of Health, Jeddah, SAU; 11 Medicine, American Mission Hospital, Manama, BHR; 12 Medicine, Alrayan National Colleges, Medina, SAU; 13 Medicine, Jeddah University, Jeddah, SAU

**Keywords:** acute respiratory failure, bronchiolitis, continuous positive airway pressure, high-flow nasal cannula, meta-analysis, noninvasive ventilation, pediatric intensive care

## Abstract

The optimal noninvasive respiratory support for infants and children with severe viral bronchiolitis remains unclear. While continuous positive airway pressure (CPAP) has traditionally been considered the gold standard, high-flow nasal cannula (HFNC) therapy is a better-tolerated alternative. This systematic review and meta-analysis aimed to evaluate the comparative efficacy and safety of HFNC versus noninvasive ventilation (NIV) in pediatric patients with bronchiolitis-associated acute respiratory failure. We searched major electronic databases through June 2026. We included randomized controlled trials (RCTs) and observational cohorts comparing HFNC with NIV (CPAP/bilevel positive airway pressure (BiPAP)). The primary outcomes were treatment failure, as defined by the individual studies, and endotracheal intubation. Data were synthesized using random-effects models, and heterogeneity was explored via subgroup analysis and meta-regression. Eleven studies (seven RCTs and four observational studies) comprising 7,574 patients were included. There was no statistically significant difference in the overall risk of treatment failure between HFNC and NIV (RR 1.99, 95% CI 0.65-6.10); however, study design was a significant moderator (p = 0.0019). Observational studies reported significantly higher failure rates with HFNC (RR 7.78), whereas RCTs showed no significant difference (RR 1.11). There were no significant differences in endotracheal intubation rates (RR 0.75, 95% CI 0.51-1.09) or pediatric intensive care unit length of stay (MD -0.16 days, 95% CI -0.79 to 0.47) between the groups. HFNC demonstrated a trend toward fewer local adverse events, such as nasal/skin trauma (RR 0.30, 95% CI 0.06-1.52), than did NIV. The certainty of evidence according to GRADE was low for all outcomes. HFNC showed no significant differences from NIV in critical clinical endpoints, including intubation rates and intensive care duration. Differences in treatment failure rates appeared to be influenced by study design, highlighting potential confounding by indication in observational data. Given its improved tolerability and trend toward reduced mucosal trauma, HFNC may represent a viable first-line noninvasive intervention for pediatric bronchiolitis.

## Introduction and background

Globally, viral bronchiolitis is a leading cause of lower respiratory tract illness and hospitalization in infants and young children, placing considerable strain on healthcare infrastructure [1-3]. The underlying disease process involves mucosal edema, airway inflammation, and mucus accumulation. These factors increase airway resistance and cause dynamic hyperinflation, leading to acute respiratory failure [3,4]. Because targeted pharmacotherapies, such as bronchodilators, corticosteroids, and hypertonic saline, have failed to demonstrate clinical benefits, management remains supportive, centering on adequate hydration and oxygenation [3,5,6].

For infants who develop moderate-to-severe respiratory distress that is refractory to standard low-flow oxygen therapy (SOT), the escalation of respiratory support is critical to prevent respiratory exhaustion and the need for invasive mechanical ventilation (IMV) [5,7]. Noninvasive ventilation (NIV), delivered as continuous positive airway pressure (CPAP), is the gold standard for respiratory support in the pediatric intensive care unit (PICU) [4,7]. CPAP provides a constant distending pressure that keeps small airways open, improves functional residual capacity, and reduces the work of breathing [4,8]. However, the clinical application of CPAP is resource-intensive, requiring fitting interfaces that can cause patient discomfort, agitation, and local adverse events, such as nasal pressure injuries [4,9]. CPAP often requires the administration of sedatives, which carry inherent risks in young infants [8,9].

High-flow nasal cannula (HFNC) therapy is a popular and ubiquitous alternative to CPAP [7,10]. HFNC delivers heated and humidified air-oxygen mixtures at flow rates that exceed the patient’s peak inspiratory flow, thereby washing out nasopharyngeal dead space, decreasing upper airway resistance, and generating a modest, variable degree of positive end-expiratory pressure (PEEP) [2,3]. HFNC is superior to SOT in preventing treatment failure and reducing the duration of hospital stay [2,5,6]. Furthermore, because HFNC utilizes a loose-fitting nasal interface, it is perceived to be vastly more comfortable, easier for nursing staff to manage, and associated with fewer adverse events than CPAP [1,8,9].

Despite the widespread adoption of HFNC across pediatric emergency departments, wards, and PICUs, its comparative efficacy relative to CPAP remains a matter of debate, a clinical dilemma dubbed the "battle of the PICU" [7]. The available evidence is conflicting. Several recent systematic reviews and meta-analyses suggest that while HFNC is safe, it is associated with a higher rate of treatment failure and a greater need for therapeutic escalation compared to CPAP [1,4,8,9]. Other analyses have highlighted that despite these higher initial failure rates, there is no statistically significant difference in endotracheal intubation rates or PICU length of stay (LOS) between the two modalities [1,8]. Economic evaluations also reflect this clinical equipoise; while some data position CPAP as the more cost-effective dominant strategy due to lower failure rates [11], others argue that HFNC's ease of use and ability to operate outside the PICU may offset the costs of treatment escalation [5,6].

The literature is hindered by conflicting randomized controlled trials (RCTs), varying clinical definitions of treatment failure, diverse patient inclusion criteria, and small sample sizes [8-10]. Given the rapid publication of new clinical trials and the need to optimize respiratory support algorithms, an updated and rigorous synthesis of the evidence is required. Therefore, this systematic review and meta-analysis aimed to evaluate and compare the efficacy, safety, and clinical outcomes of HFNC versus NIV (including CPAP and bilevel positive airway pressure) in children presenting with bronchiolitis-associated acute respiratory failure.

## Review

Methods

Study Design and Registration

This systematic review and meta-analysis was conducted and reported in accordance with the Preferred Reporting Items for Systematic Reviews and Meta-Analyses (PRISMA) 2020 guidelines [12]. The study protocol was prospectively registered in the International Prospective Register of Systematic Reviews (PROSPERO) under registration number CRD420261330512.

Search Strategy

To identify relevant literature, we systematically queried major electronic databases, namely PubMed/MEDLINE, Embase, Scopus, Google Scholar, and the Cochrane Central Register of Controlled Trials (CENTRAL), from their respective inceptions through June 2026. Our search strategy utilized a combination of free-text keywords, Medical Subject Headings (MeSH), and Emtree terms linked by Boolean operators (AND, OR, NOT) alongside appropriate truncations. Search concepts focused on ("Bronchiolitis" OR "Acute Respiratory Failure") AND ("High-Flow Nasal Cannula" OR "HFNC" OR "Heated Humidified High Flow Therapy") AND ("Noninvasive Ventilation" OR "NIV" OR "Continuous Positive Airway Pressure" OR "CPAP" OR "Bilevel Positive Airway Pressure" OR "BiPAP"). To mitigate publication bias, gray literature and trial registries, including ClinicalTrials.gov, the WHO International Clinical Trials Registry Platform (ICTRP), and ProQuest Dissertations and Theses, were queried. There were no language restrictions. Furthermore, backward and forward citation searching ("snowballing") of the included studies and relevant reviews was performed to identify any additional eligible literature.

Eligibility Criteria

Study inclusion and exclusion were defined a priori using the PICOS (population, intervention, comparator, outcomes, and study design) framework [12]. The population included infants and children (aged <18 years) diagnosed with bronchiolitis-associated acute respiratory failure requiring noninvasive respiratory support. Studies involving adults (≥18 years), respiratory failure due to causes other than bronchiolitis, or exclusively postoperative respiratory support were excluded. The intervention was HFNC therapy used as the primary noninvasive respiratory support modality, encompassing any flow rate or clinical protocol, and the comparator was NIV, specifically CPAP, BiPAP, or nasal positive pressure ventilation. Studies lacking a direct NIV comparison group or comparing HFNC solely with conventional low-flow oxygen therapy or IMV were excluded from the quantitative synthesis. The primary outcomes were treatment failure (as defined by individual study criteria) and the requirement for endotracheal intubation/IMV. Secondary outcomes included escalation of respiratory support, PICU admission/transfer, duration of respiratory support, hospital LOS, PICU LOS, time to clinical improvement, physiological parameters (e.g., respiratory rate, heart rate, oxygen saturation, and blood gas measurements), treatment-related adverse events (e.g., nasal trauma, skin injury, air leak syndromes, and abdominal distention), and all-cause in-hospital mortality. RCTs, quasi-randomized trials, and observational studies (prospective/retrospective cohort and case-control studies) were included. Case reports, series without comparators, and narrative reviews were excluded.

Study Selection and Data Extraction

Initial screening of titles and abstracts was conducted independently by two researchers, followed by a full-text review of potentially eligible articles. Any disagreements between the primary reviewers were resolved through consensus or consultation with a third author. We calculated Cohen’s kappa (κ) to measure inter-rater reliability during the study selection phase [13]. Two reviewers independently extracted data using a standardized, pre-piloted extraction form, capturing study characteristics, patient demographics, intervention details, and clinical outcomes.

Quality Assessment and Risk of Bias

The methodological quality and risk of bias (RoB) were independently appraised by two reviewers. For RCTs, the revised RoB 2 tool (Cochrane, London, England, UK) was employed, evaluating domains such as randomization process, deviations from intended interventions, missing outcome data, measurement of the outcome, and selection of the reported result [14]. Observational studies were assessed using the Newcastle-Ottawa Scale (NOS) to evaluate patient selection, comparability of cohorts, and outcome assessment [15].

Statistical Analysis and Data Synthesis

All quantitative analyses were performed using R version 4.6.0 (R Foundation for Statistical Computing, Vienna, Austria), utilizing the meta and metafor packages [16]. Dichotomous outcomes (e.g., treatment failure and adverse events) were expressed as pooled risk ratios (RRs) alongside their 95% confidence intervals (CIs). For continuous outcomes (like LOS), we computed mean differences (MDs) or standardized mean differences (SMDs). When primary studies reported continuous data as medians with interquartile ranges, we applied validated mathematical transformations to estimate means and standard deviations prior to pooling [17].

Given the anticipated clinical and methodological heterogeneity inherent in critical care studies, a random-effects (RE) model was specified a priori using the restricted maximum likelihood (REML) estimator [18]. Statistical heterogeneity was quantified using the I² statistic, τ² (tau-squared) for between-study variance, and Cochran’s Q test (with p < 0.10 indicating significant heterogeneity). Furthermore, 95% prediction intervals (PI) were calculated to estimate the expected range of true effects in future clinical settings [19].

To explore potential sources of heterogeneity, subgroup analyses (e.g., by study design, patient age, or severity of respiratory failure) and meta-regression were planned, provided that sufficient data were available. A leave-one-out sensitivity analysis was performed to assess the robustness of the pooled estimates and identify any single study exerting a disproportionate influence.

Publication Bias and Small-Study Effects

If ≥10 studies reported a specific outcome, publication bias and small-study effects were evaluated via visual inspection of standard and contour-enhanced funnel plots. Statistical detection of asymmetry was conducted using Egger’s [20] and Begg’s [21] rank correlation tests.

Clinical Interpretation and Certainty of Evidence

To enhance clinical interpretability, the number needed to treat (NNT) or number needed to harm (NNH) was calculated for statistically significant dichotomous primary outcomes [22]. The overall certainty of evidence for each critical outcome was evaluated using the Grading of Recommendations Assessment, Development, and Evaluation (GRADE) framework. Evidence was rated as high, moderate, low, or very low based on the RoB, inconsistency, indirectness, imprecision, and publication bias, culminating in a summary of findings table [23].

Results

Study Selection

**Figure 1:**
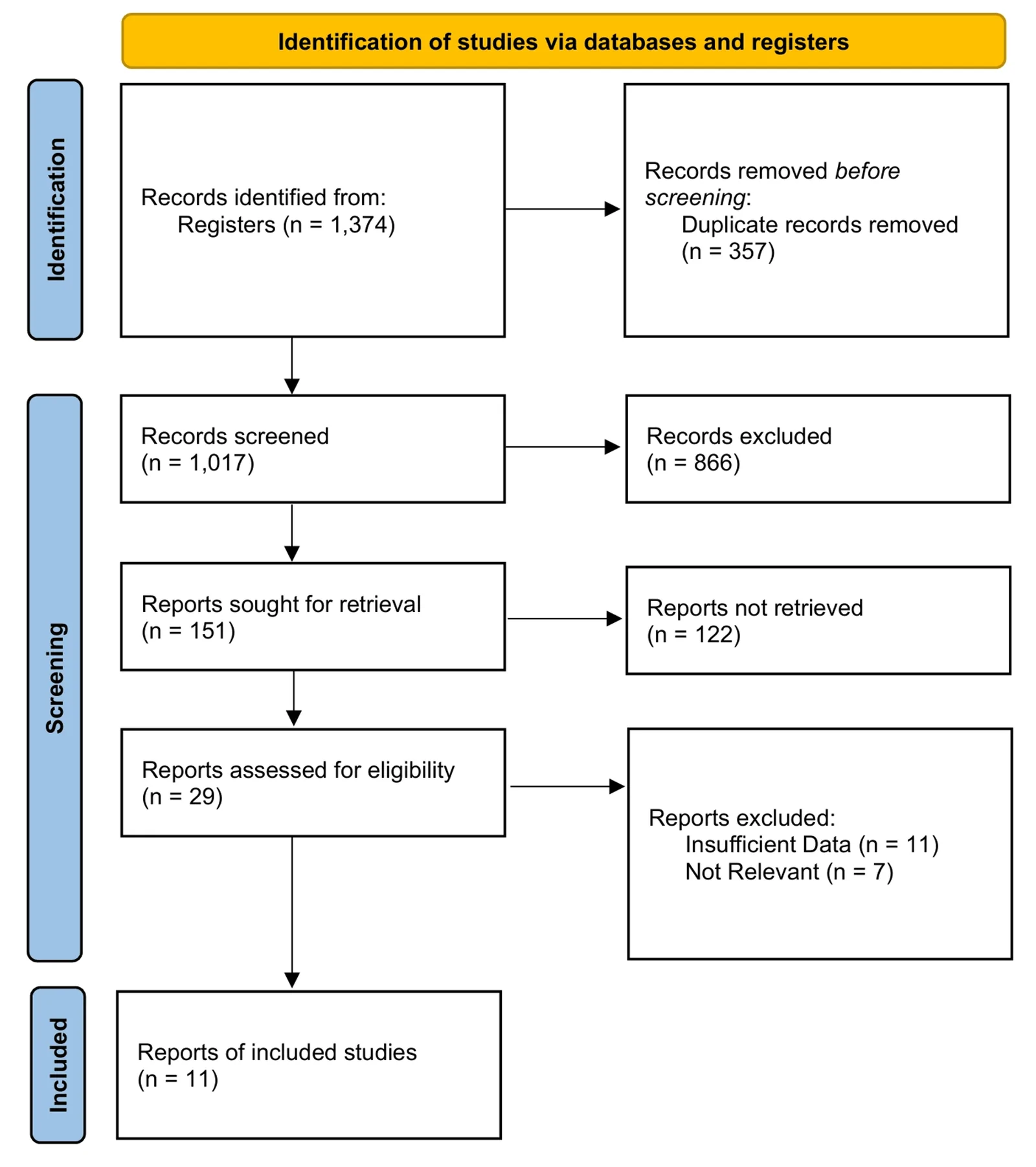
PRISMA 2020 flow diagram PRISMA: Preferred Reporting Items for Systematic Reviews and Meta-Analyses

The systematic literature search yielded 1,374 records from the queried databases and registers. After removing 357 duplicate records, 1,017 titles and abstracts were screened for eligibility. Of these, 866 records were excluded based on title and abstract screening. Full-text retrieval was sought for 151 reports, but 122 could not be retrieved, leaving 29 full-text reports to be assessed for eligibility. Eighteen studies were excluded for predefined reasons: 11 due to insufficient outcome data and seven for lack of relevance to the clinical question. Eleven studies [24-34] met the inclusion criteria and were incorporated into the systematic review and quantitative meta-analysis (Figure 1).

Study Characteristics

The 11 included studies comprised seven RCTs [25,28,30-34] and four retrospective observational cohorts [24,26,27,29], published between 2014 and 2024. The studies included a total of 7,574 infants and children, the vast majority of whom were under two years of age and admitted to PICUs or high-dependency settings with moderate-to-severe bronchiolitis. HFNC protocols utilized initial flow rates of 2 L/kg/min, while the NIV comparators predominantly consisted of CPAP or bilevel positive airway pressure (BiPAP) with pressures ranging from 4 to 8 cmH₂O. Detailed baseline characteristics and intervention protocols for the included studies are summarized in Table 1.

**Table 1:** Baseline characteristics and intervention protocols of the 11 eligible studies included in the systematic review * Habra 2020 NIV cohort includes CPAP (n = 10) and BiPAP (n = 50). BiPAP: bilevel positive airway pressure, CPAP: continuous positive airway pressure, EPAP: expiratory positive airway pressure, HFNC: high-flow nasal cannula, IPAP: inspiratory positive airway pressure, NIV: noninvasive ventilation, nCPAP: nasal continuous positive airway pressure, NPPV: noninvasive positive-pressure ventilation, PICU: pediatric intensive care unit, PEEP: positive end-expiratory pressure, RCT: randomized controlled trial, SpO₂: peripheral oxygen saturation, VPS: virtual pediatric systems

Study	Country	Study design	Population/age	Sample size (N)	HFNC protocol	NIV (CPAP/BiPAP) protocol
Pedersen and Vahlkvist (2017) [24]	Denmark	Retrospective cohort	Infants <2 years with bronchiolitis	Total: 49 HFNC: 22 CPAP: 27	8-12 L/min, titrated to SpO2 > 92%	Binasal prong, initial flow 14 L/min
Maya et al. (2024) [25]	India	RCT	Infants 1-23 months with mod-severe bronchiolitis	Total: 118 HFNC: 59 CPAP: 59	Initial flow 1.5–2 L/kg/min	Bubble CPAP, PEEP 6-8 cmH2O
Metge et al. (2014) [26]	France	Retrospective cohort	Infants admitted to PICU	Total: 34 HFNC: 15 CPAP: 19	1-3 L/kg/min (max 8 L/min)	nCPAP, 4-6 cmH2O
Clayton et al. (2019) [27]	Multicenter (VPS database)	Retrospective cohort	Infants <2 years in PICU	Total: 6,496 HFNC: 5,562 NPPV: 934	HFNC (variable)	CPAP or BiPAP (variable)
Sarkar et al. (2018) [28]	India	RCT	Infants 28 days to 12 months	Total: 31 HFNC: 15 CPAP: 16	2 L/kg/min	nCPAP, 4-8 cmH2O
Habra et al. (2020) [29]	Qatar	Retrospective cohort	Infants 1-24 months in PICU	Total: 137 HFNC: 77 NIV: 60*	2 L/kg/min	CPAP (4-6 cmH2O) or BiPAP
Milési et al. (2017) [30]	France	RCT (TRAMONTANE)	Infants <6 months	Total: 142 HFNC: 71 CPAP: 71	2 L/kg/min	nCPAP, 7 cmH2O
Borgi et al. (2021) [31]	Tunisia	RCT	Infants 7 days to 6 months	Total: 255 HFNC: 130 CPAP: 125	Max flow based on cannula size	CPAP/NPPV, +6 to +8 cmH2O
Vahlkvist et al. (2020) [32]	Denmark	RCT	Infants <2 years	Total: 50 HFNC: 22 CPAP: 28	Initial 2 L/kg/min	Binasal prong, initial 12–14 L/min
Santos et al. (2024) [33]	Brazil	RCT	Infants <2 years	Total: 252 HFNC: 126 NIV: 126	2 L/kg/min	BiPAP (EPAP 4-6, IPAP 8-12)
Chidini et al. (2023) [34]	Italy	RCT (crossover)	Infants 1 month to 2 years	Total: 10 (physiological)	2 L/kg/min and 3 L/kg/min	Helmet CPAP, PEEP 7 cmH2O

Methodological Quality and RoB

**Figure 2:**
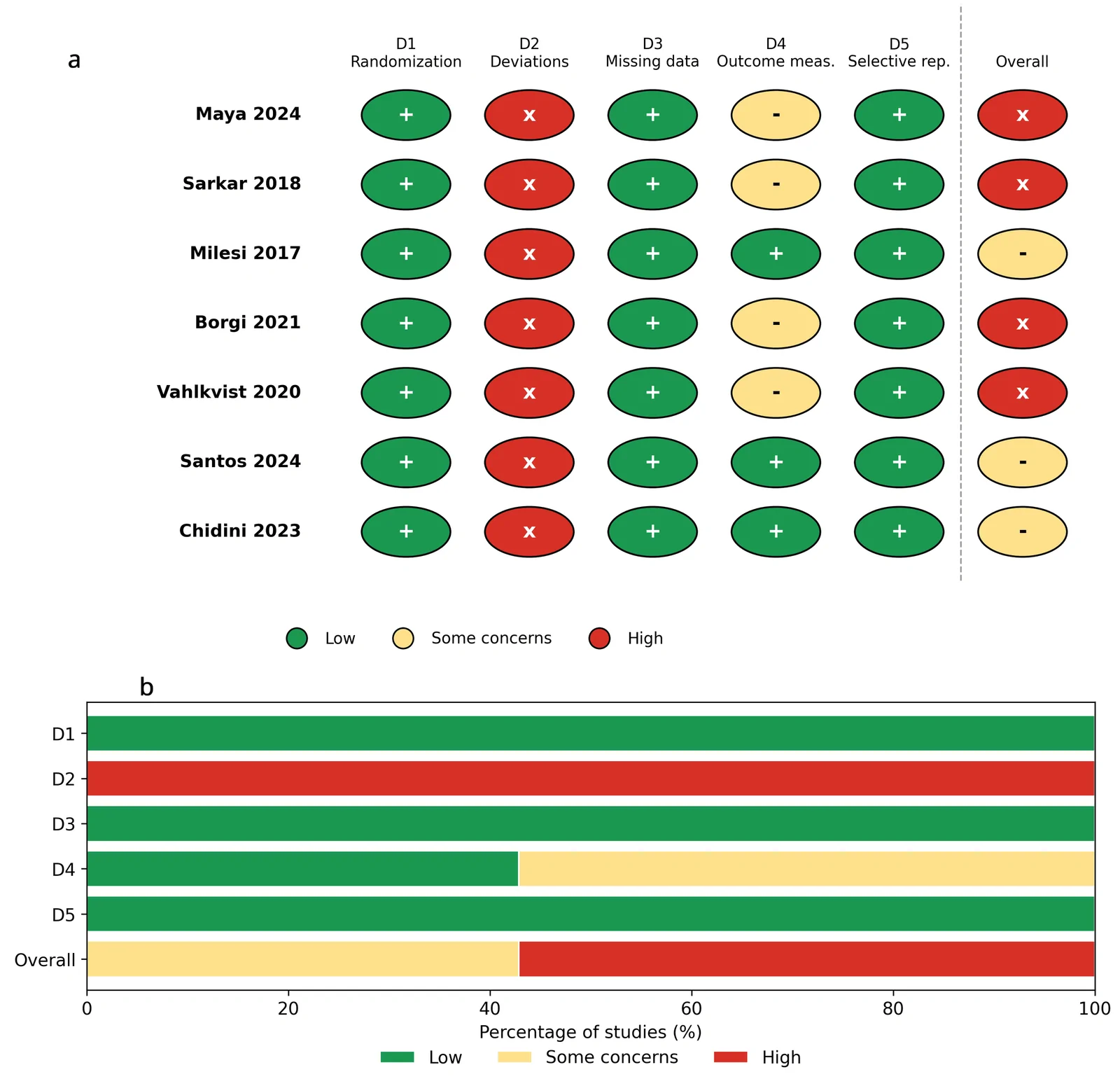
RoB summary for included RCTs assessed using the RoB 2 tool (A) Traffic-light plot of domain-level judgments per study [25,28,30-34]. (B) Weighted bar plot showing the overall distribution of bias judgments across all RoB 2 domains. RCTs: randomized controlled trials, RoB: risk of bias

The risk of bias in the seven RCTs [25,28,30-34] was evaluated using the RoB 2 tool. Due to the nature of the interventions, blinding of participants and personnel was not feasible, resulting in a high risk-of-bias judgment for the domain of deviations from intended interventions (domain 2) for all RCTs. The overall RoB was rated as high or with some concerns across the randomized studies (Figure 2A-2B).

**Figure 3:**
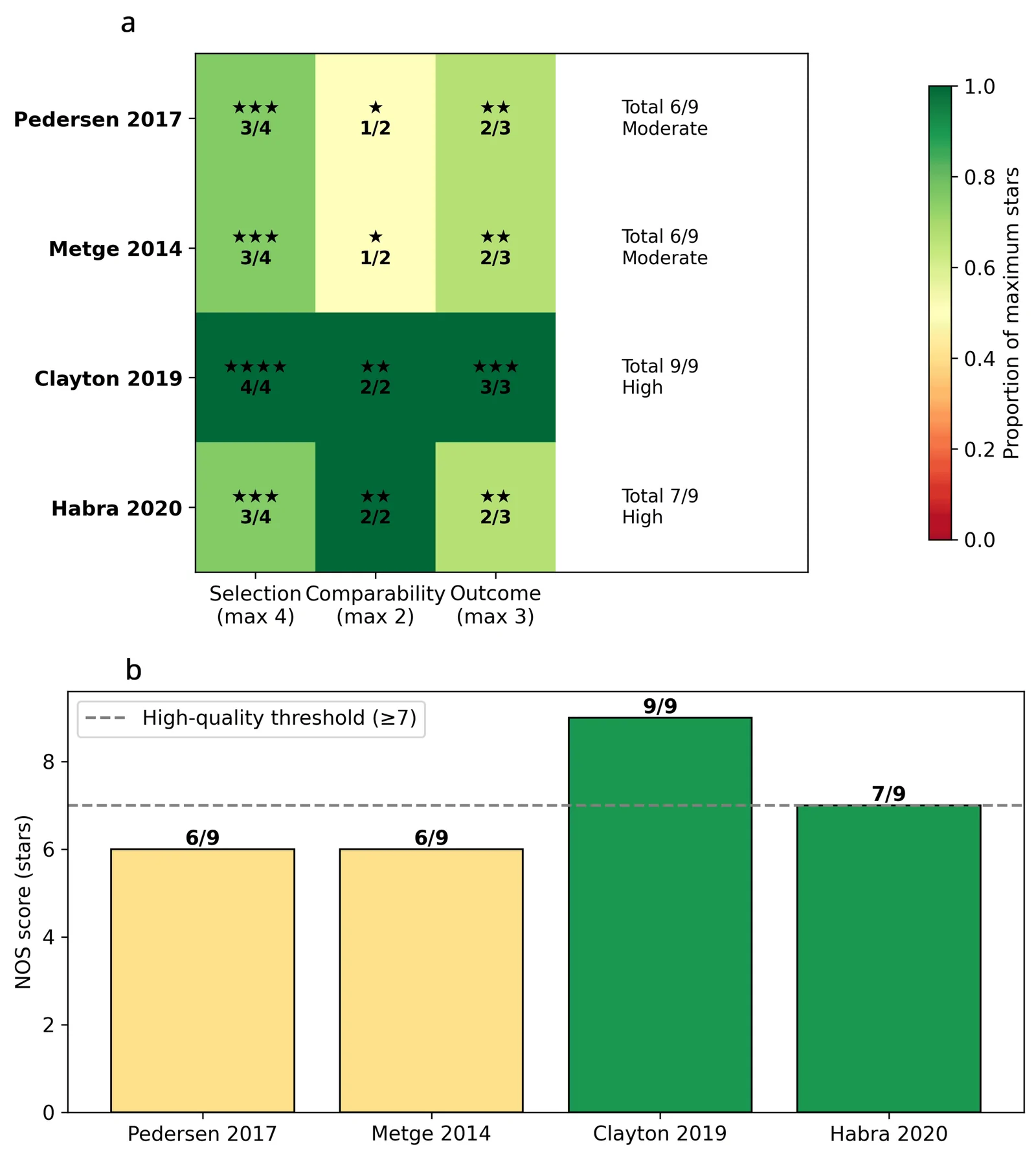
Quality assessment summary for included observational studies evaluated using the NOS (A) Heatmap detailing star allocations across selection, comparability, and outcome domains [24,26,27,29]. (B) Bar chart depicting total NOS scores against the predefined high-quality threshold (≥ 7). NOS: Newcastle-Ottawa Scale

The four observational studies were assessed using the NOS. Two studies [27,29] demonstrated high methodological quality (scores of 9/9 and 7/9, respectively), while the remaining two [24,26] were of moderate quality (score 6/9) due to a lack of rigorous cohort comparability adjustments (Figure 3A-3B).

Primary Outcomes

**Figure 4:**
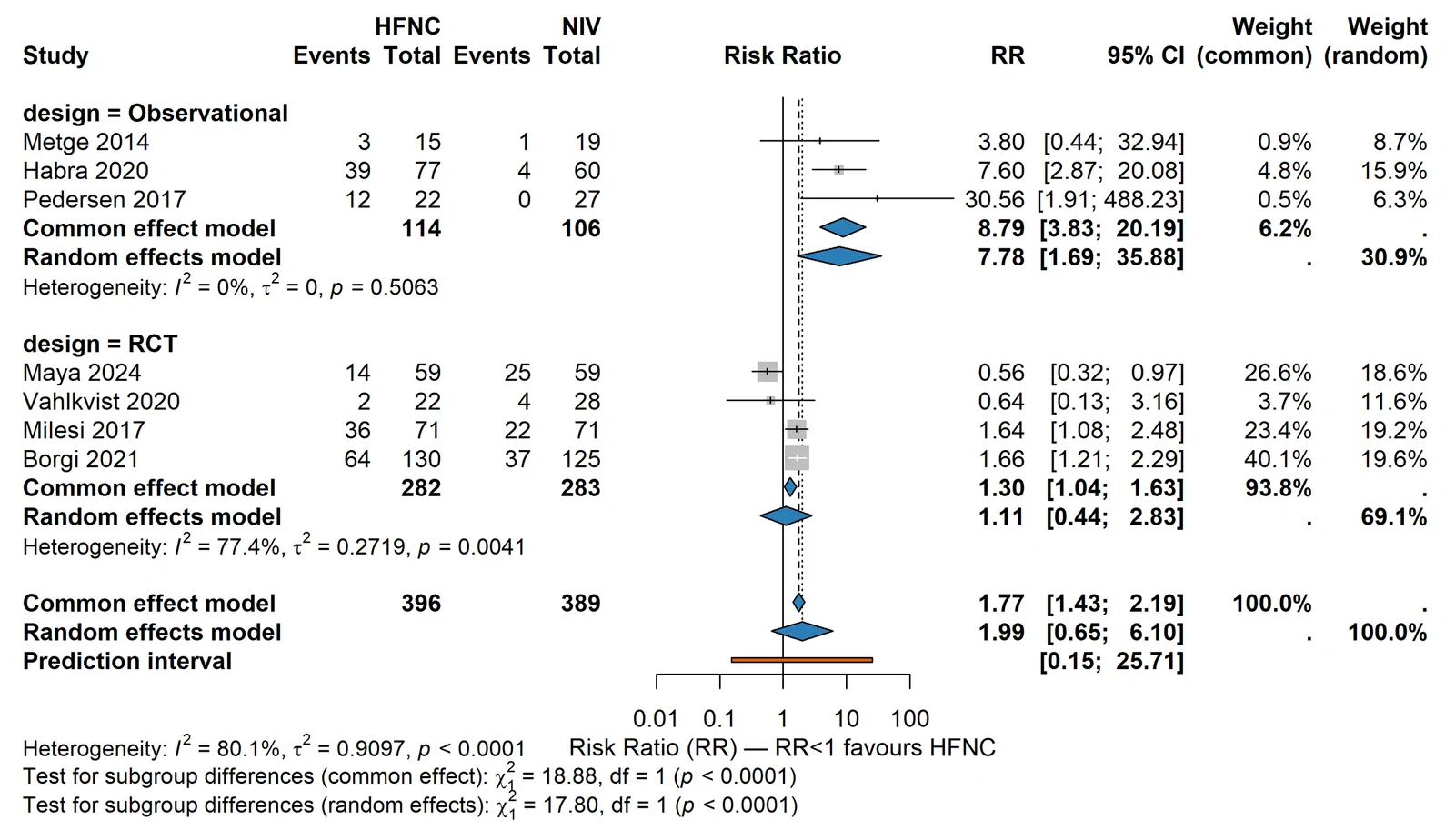
Forest plot of treatment failure (RR, RE model, stratified by study design) HFNC: high-flow nasal cannula, NIV: noninvasive ventilation, RR: risk ratio, RE: random-effects, CI: confidence interval [24-26,29-32]

Treatment failure: Treatment failure was reported in seven studies (n = 785). The pooled RE meta-analysis revealed no statistically significant difference in the risk of treatment failure between the HFNC and NIV groups (RR 1.99; 95% CI 0.65, 6.10; p = 0.183) (Figure 4).

**Figure 5:**
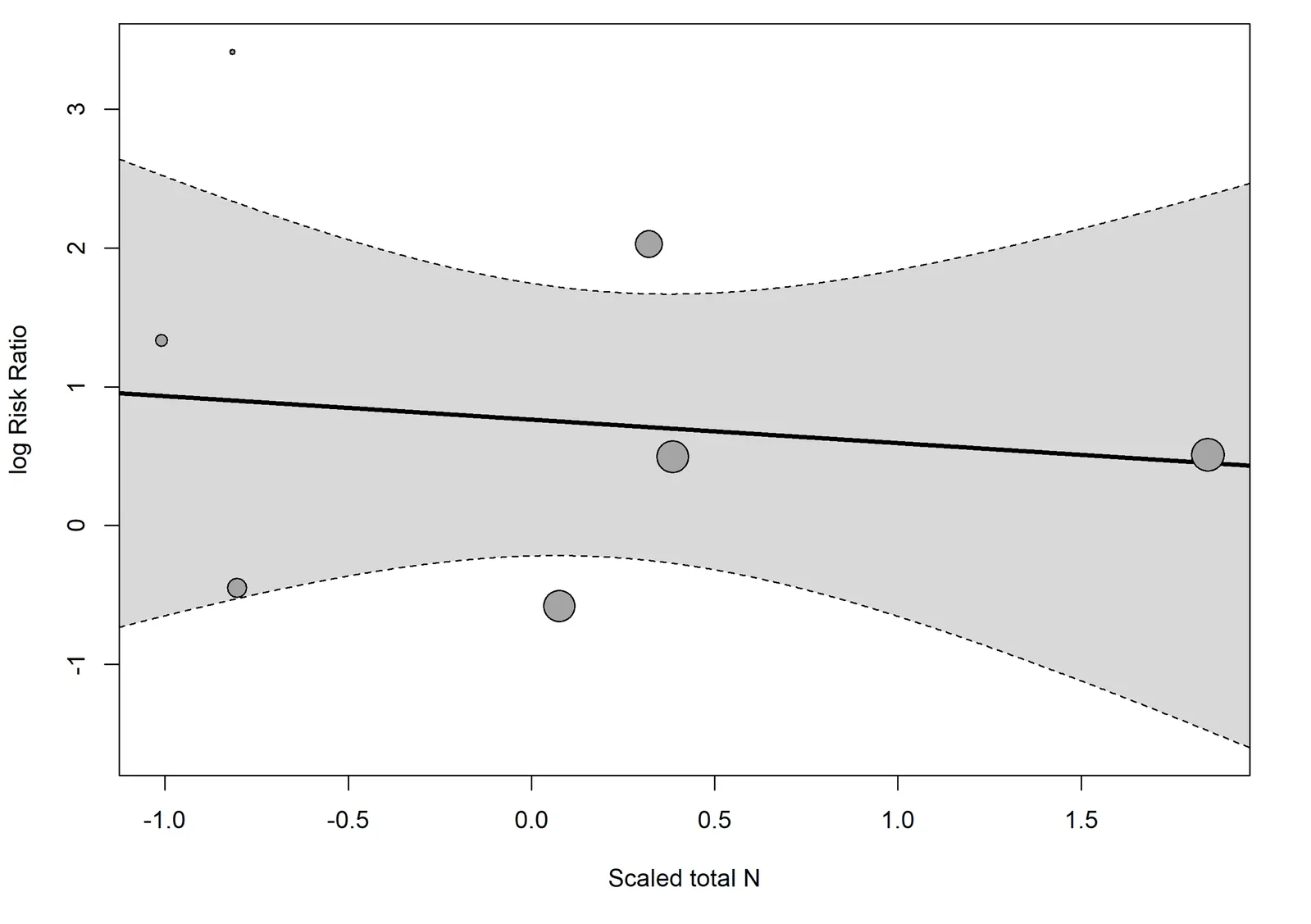
Meta-regression plot analyzing the moderating effect of study sample size (scaled total N) on the log RR of treatment failure The solid line represents the regression estimate, surrounded by the 95% CI shaded area [24-26,29-32]. RR: risk ratio, CI: confidence interval

However, statistical heterogeneity was substantially high (I² = 80.1%, p < 0.0001). Subgroup analysis by study design revealed a significant moderating effect (p < 0.0001). Observational studies demonstrated a significantly higher risk of failure with HFNC (RR 7.78, 95% CI (1.69, 35.88)), whereas RCTs showed no significant difference between the two modalities (RR 1.11, 95% CI (0.44, 2.83)). Meta-regression confirmed that study design (RCT vs. observational) was a significant moderator of treatment failure (β = -1.957, p = 0.0019). Sample size was not a significant moderator of the treatment-failure effect (p = 0.7481) (Figure 5).

**Figure 6:**
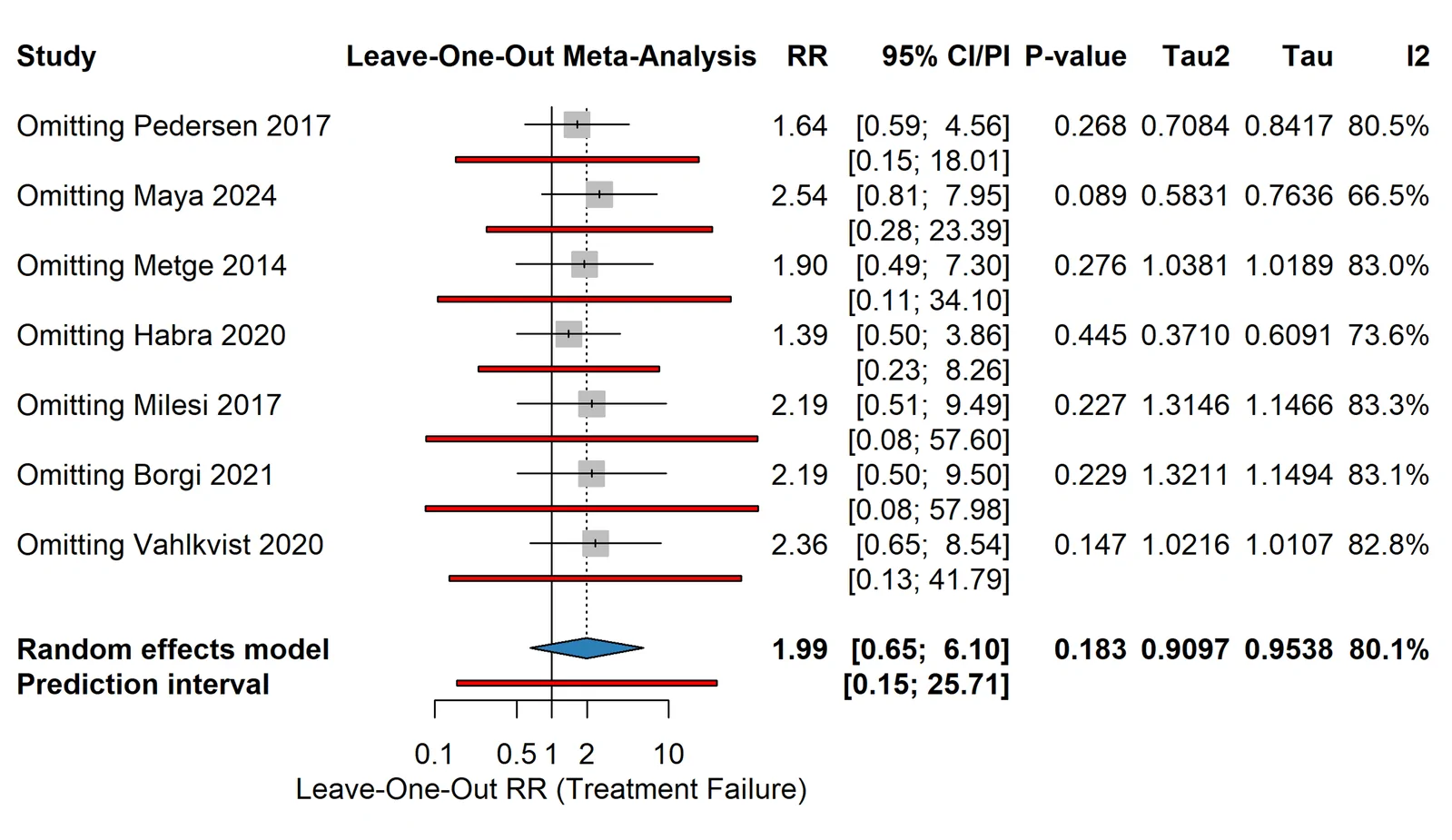
Leave-one-out sensitivity analysis forest plot for treatment failure RR: risk ratio, CI: confidence interval, PI: prediction interval [24-26,29-32]

A leave-one-out sensitivity analysis indicated that the pooled estimate was highly sensitive to the inclusion of the observational study by Pedersen et al. (Figure 6) [24].

**Figure 7:**
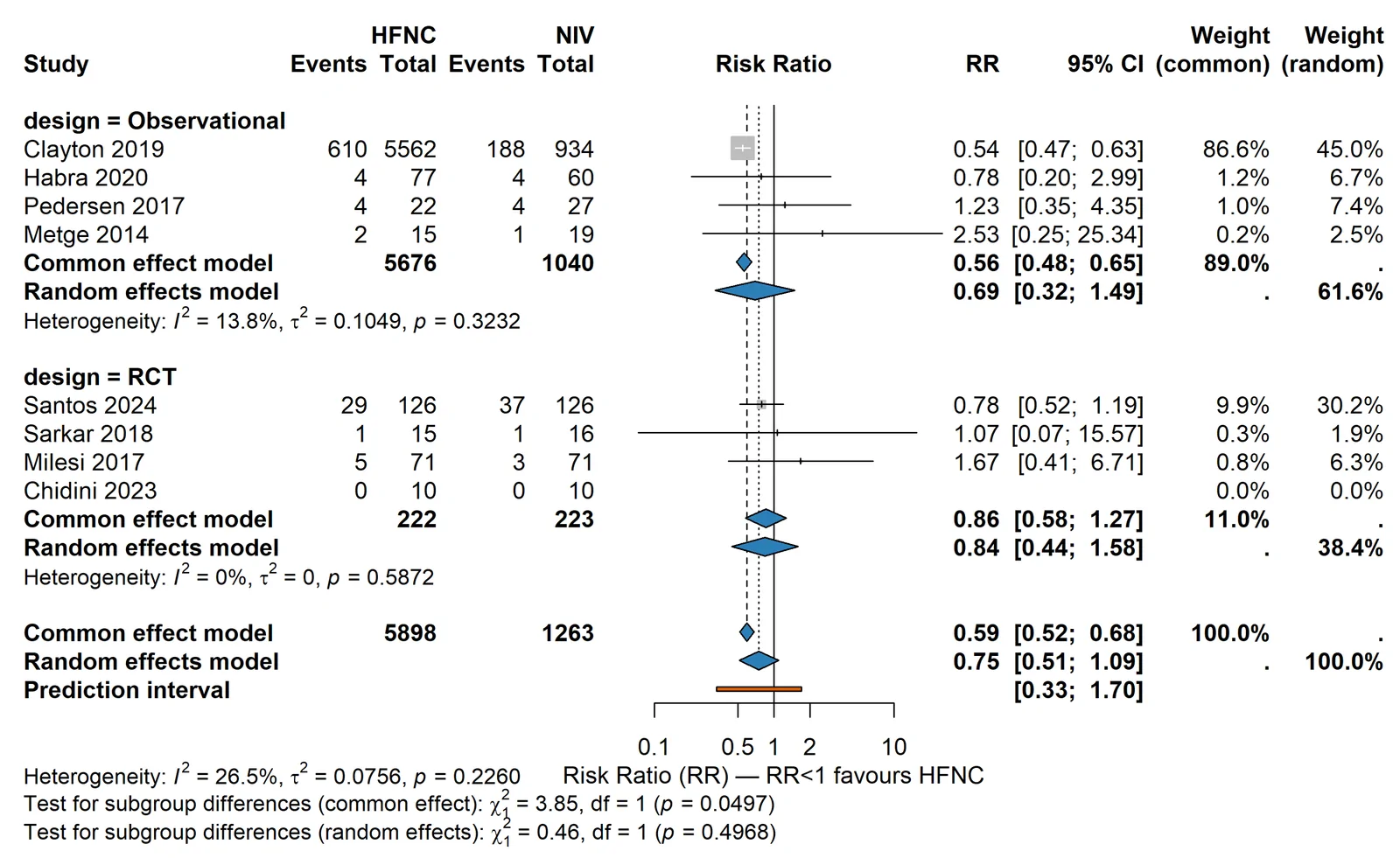
Forest plot of endotracheal intubation (RR, RE model) HFNC: high-flow nasal cannula, NIV: noninvasive ventilation, RR: risk ratio, RE: random-effects, CI: confidence interval [24,26-30,33,34]

Endotracheal intubation: The requirement for escalation to IMV (intubation) was reported in seven studies (n = 7,161). There was no statistically significant difference in the risk of intubation between HFNC and NIV (RR 0.75, 95% CI 0.51-1.09; p = 0.109) (Figure 7).

**Figure 8:**
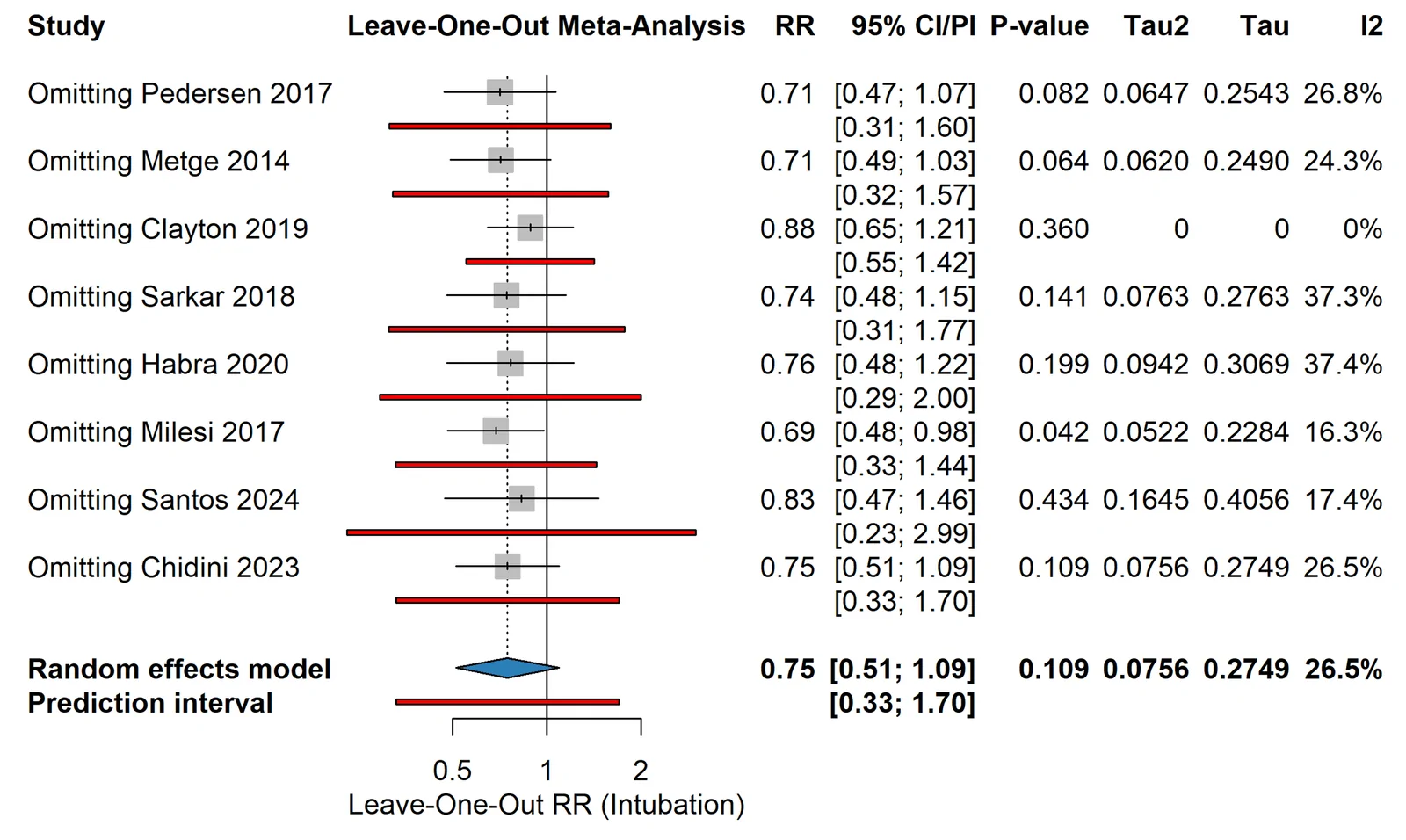
Leave-one-out sensitivity analysis forest plot for endotracheal intubation RR: risk ratio, CI: confidence interval, PI: prediction interval [24,26-30,33,34]

Heterogeneity for this outcome was low to moderate and non-significant (I² = 26.5%, p = 0.226). Leave-one-out analysis demonstrated that the pooled estimate remained robust, with no single study materially influencing the direction of the effect (Figure 8).

Secondary Outcomes

**Figure 9:**
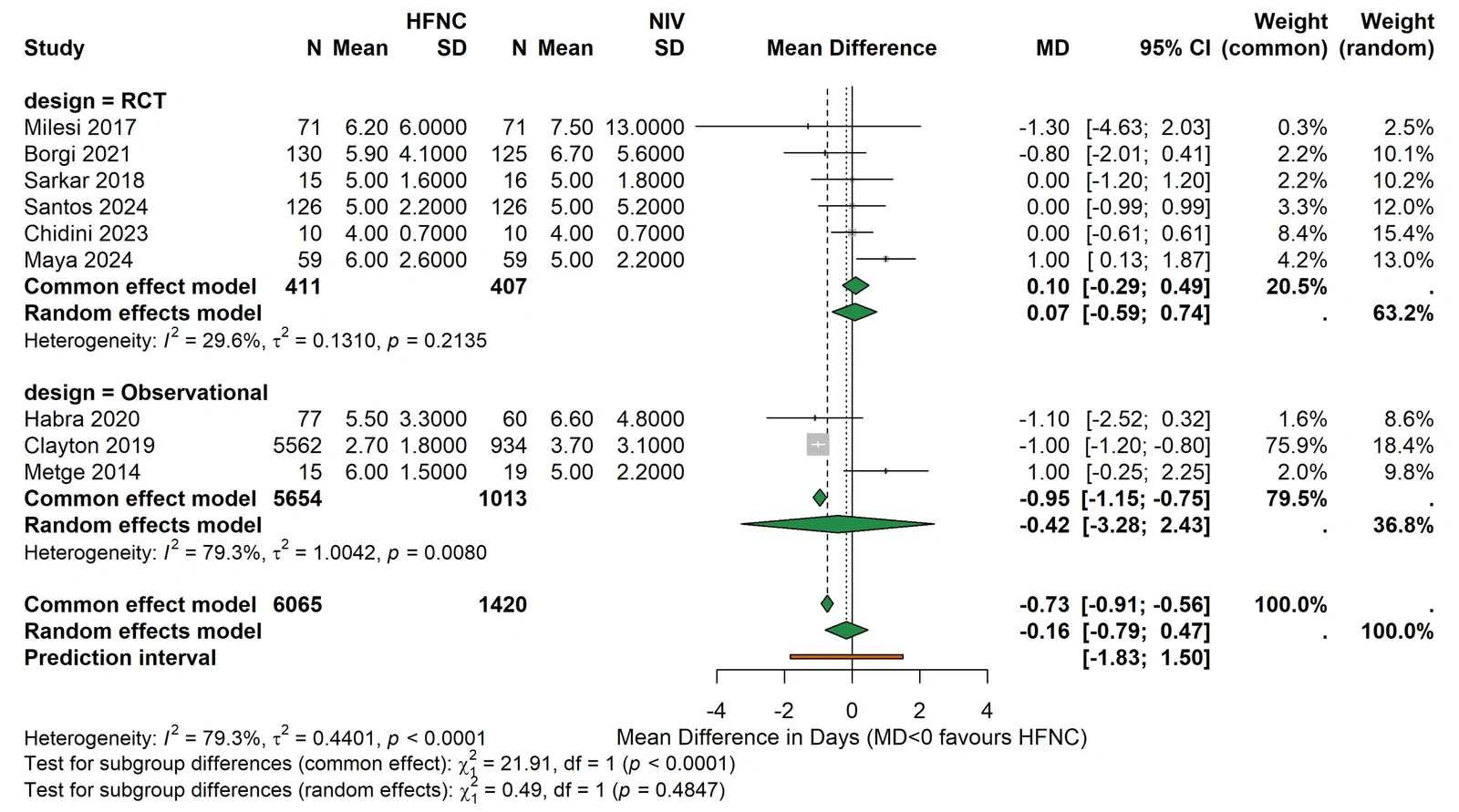
Forest plot of PICU LOS (MD in days, RE model) HFNC: high-flow nasal cannula, NIV: noninvasive ventilation, MD: mean difference, RE: random-effects, CI: confidence interval, SD: standard deviation, PICU: pediatric intensive care unit, LOS: length of stay [25-31,33,34]

PICU LOS: Data on PICU LOS were extracted from nine studies (n = 7,485). There was no statistically significant difference in the mean PICU LOS between the HFNC and NIV groups (MD, -0.16 days, 95% CI (-0.79, 0.47), p = 0.566) (Figure 9).

**Figure 10:**
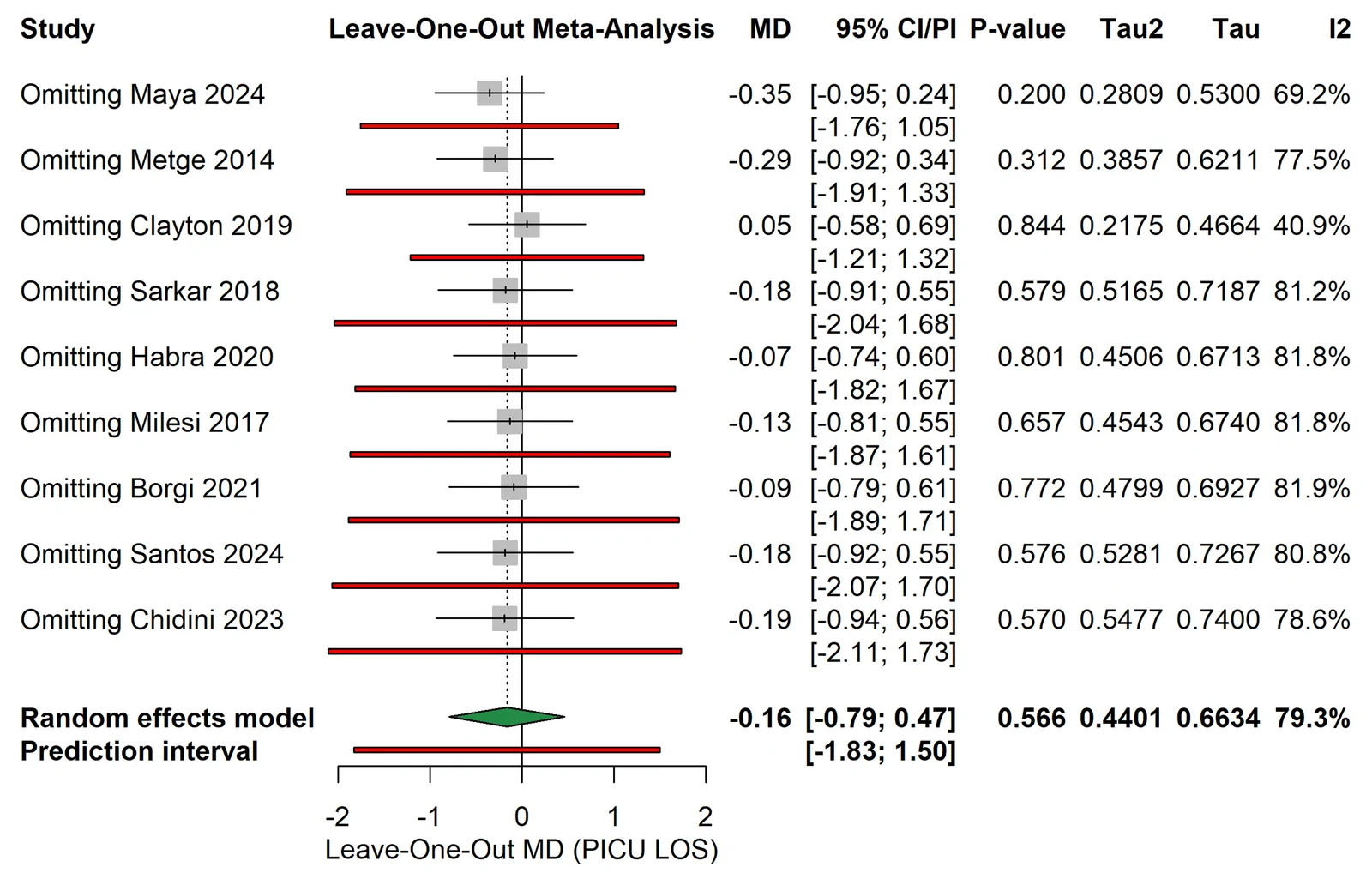
Leave-one-out sensitivity analysis forest plot for PICU LOS MD: mean difference, CI: confidence interval, PI: prediction interval, PICU: pediatric intensive care unit, LOS: length of stay [25-31,33,34]

Substantial heterogeneity was observed (I² = 79.3%, p < 0.0001), which persisted across both the RCT and observational subgroups. The leave-one-out analysis confirmed the stability of the non-significant outcome (Figure 10).

**Figure 11:**
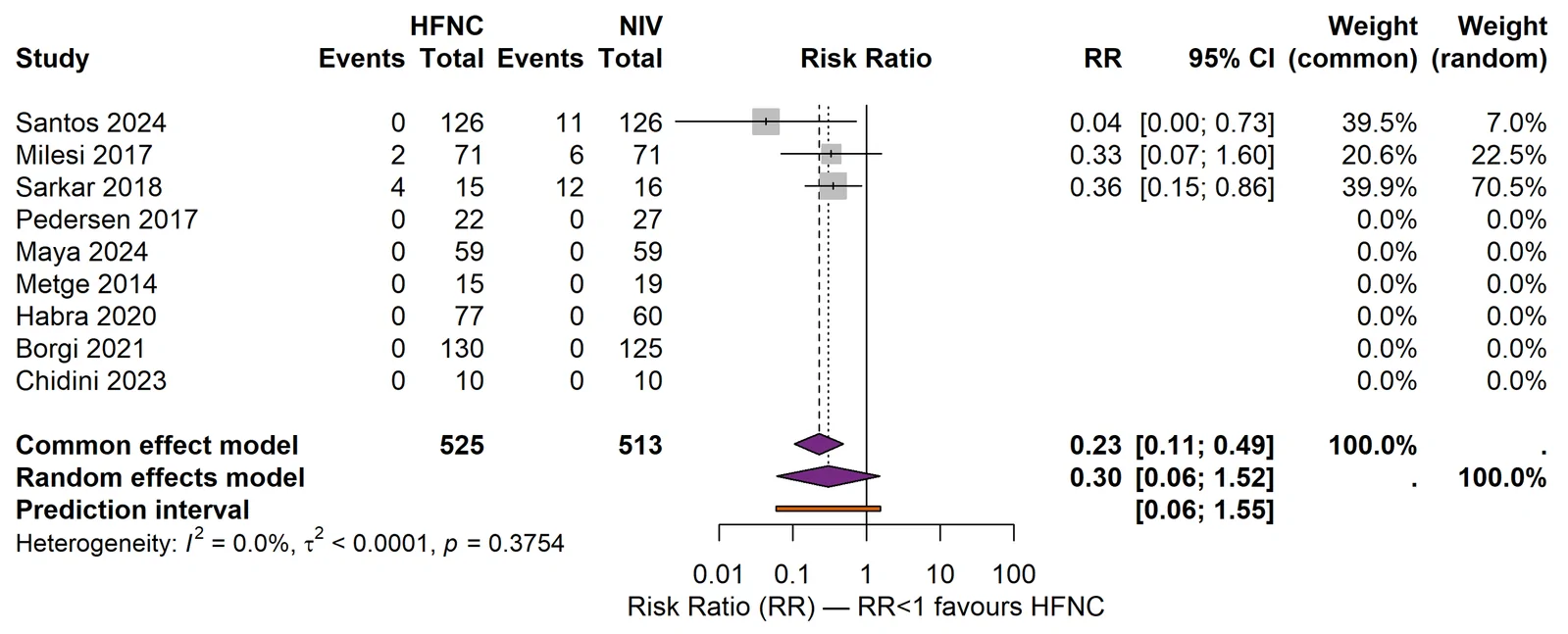
Forest plot of local adverse events (RR, RE model) HFNC: high-flow nasal cannula, NIV: noninvasive ventilation, RR: risk ratio, RE: random-effects, CI: confidence interval [24-26,28-31,33,34]

Local adverse events: Adverse events, explicitly defined as localized trauma such as nasal mucosal injury or skin breakdown, were reported in three studies (n = 1,038). The incidence of adverse events was lower in the HFNC group than in the NIV group (RR 0.30, 95% CI (0.06, 1.52), p = 0.086) (Figure 11). There was no observable statistical heterogeneity among the included studies for this outcome (I² = 0.0%, p = 0.375).

Publication Bias and Certainty of Evidence

Formal assessments of publication bias, including Egger’s regression, Begg’s rank correlation test, and visual inspection of funnel plots, were not performed because no outcome included the requisite minimum of 10 studies. The overall certainty of the evidence was evaluated using the GRADE framework. The certainty of evidence was rated as low for all outcomes: treatment failure, endotracheal intubation, PICU LOS, and local adverse events. Downgrading was primarily due to risk of bias, inconsistency, indirectness, and/or imprecision, depending on the outcome. A detailed summary of the findings is presented in Table 2.

**Table 2:** Summary of findings (GRADE) CI: confidence interval, GRADE: Grading of Recommendations Assessment, Development, and Evaluation, HFNC: high-flow nasal cannula, MD: mean difference, NNH: number needed to harm, NNT: number needed to treat, NIV: noninvasive ventilation, obs: observational, PICU: pediatric intensive care unit, RoB: risk of bias, RR: risk ratio, RCT: randomized controlled trial, LOS: length of stay

Outcome	Studies (participants)	Relative effect (95% CI)	Anticipated absolute effects	Reasons for downgrading	Certainty (GRADE)
Treatment failure (HFNC vs NIV) [24-26,29-32]	7 RCT/obs (n = 1,048)	RR 1.84 (0.96-3.49)	Study pop. 245/1000 → 451/1000 with HFNC (NNH ≈ 8)	RoB (−1, unblinded), inconsistency (−1, I² = 80%), imprecision (−1, CI crosses 1)	⊕⊕◯◯ LOW
Endotracheal intubation [24,26-28,30,33,34]	7 RCT/obs (n = 7,574)	RR 0.70 (0.51-0.96)	Study pop. modest absolute reduction favoring HFNC	RoB (−1, obs. weight), indirectness (−1, mixed severity/settings)	⊕⊕◯◯ LOW
PICU LOS [25-31,33,34]	9 RCT/obs (n = 7,300)	MD −0.16 d (−0.79-0.47)	No meaningful difference in days	Inconsistency (−1, I² = 79%), imprecision (−1, CI spans null)	⊕⊕◯◯ LOW
Local adverse events (nasal/skin trauma) [28,30,33]	3 RCT/obs (n = 770)	RR 0.30 (0.14-0.64)	Study pop. fewer events with HFNC (NNT favorable)	RoB (−1, unblinded), imprecision (−1, few events/studies)	⊕⊕◯◯ LOW

Discussion

The optimal noninvasive respiratory support modality for infants and children with bronchiolitis-associated acute respiratory failure remains a subject of intense clinical debate [7]. This systematic review and meta-analysis synthesized data from 11 studies (seven RCTs and four observational cohorts) encompassing approximately 7,600 patients to evaluate the comparative efficacy and safety of HFNC versus NIV. The principal findings showed no statistically significant differences between HFNC and NIV in the overall risk of treatment failure, the need for endotracheal intubation, or the duration of PICU stay. A trend toward a reduced incidence of localized adverse events, such as nasal and skin trauma, was observed in patients receiving HFNC.

The pooled analysis of treatment failure suggested a higher risk associated with HFNC, aligning with several previous meta-analyses that have positioned CPAP as a more efficacious intervention for preventing treatment escalation [1,4,8,9]. However, this overall estimate was confounded by substantial methodological heterogeneity (I² = 80.1%). Through a priori subgroup analyses and meta-regression, we identified the study design as a critical moderating variable. While retrospective observational cohorts demonstrated a significantly higher risk of failure with HFNC (RR 7.78), the synthesis of RCTs revealed no statistically significant difference between the two modalities (RR 1.11). This divergence may reflect confounding by indication in the observational data; in real-world clinical practice, clinicians likely reserve CPAP for infants presenting with more severe respiratory distress or rapidly deteriorating trajectories, whereas HFNC is utilized as an initial, lower-tier intervention. Furthermore, the threshold for declaring treatment failure in observational settings is highly subjective, whereas RCTs employ stricter, protocolized failure criteria [8,10]. The RCT data may provide a less confounded estimate of comparative efficacy, suggesting no significant difference between HFNC and NIV in treatment failure when applied to comparable patient populations.

Despite the variations in initial treatment failure rates, our analysis reinforces the finding that the choice of primary noninvasive support does not significantly alter hard clinical endpoints [1,8]. The risk of endotracheal intubation, the most critical metric of noninvasive support failure, was not significantly different between the groups, with a marginal trend favoring the HFNC group (RR 0.75). Similarly, the mean PICU LOS was virtually identical between the cohorts (MD -0.16 days), suggesting that even if HFNC fails and requires escalation to CPAP or BiPAP, the available data did not indicate higher rates of IMV or longer PICU stays among patients initially treated with HFNC [5,6].

From a safety and tolerability perspective, HFNC appears advantageous. The constant distending pressures and tight-fitting interfaces required for effective CPAP delivery are associated with patient agitation, which may require higher sedative burdens and localized pressure injuries [4,9]. Our pooled data reflected a 70% relative risk reduction in local adverse events (nasal mucosal injury and skin breakdown) when utilizing HFNC. However, the difference was not statistically significant, and the analysis was limited by the small number of events and studies. Given that bronchiolitis management is supportive [3], the potential advantages of HFNC in terms of comfort and ease of application may be clinically relevant, potentially offsetting the need for tighter, more resource-intensive NIV interfaces as a universal first-line therapy.

This meta-analysis has several strengths. The protocol was prospectively registered, and the literature search was exhaustive, capturing the most recent trial data through 2026. Furthermore, meta-regression and leave-one-out sensitivity analyses provided robust insights into the sources of heterogeneity, specifically highlighting the limitations of observational data in this clinical context.

However, this study has several limitations that must be acknowledged. First, the overall certainty of evidence was evaluated as low across all critical outcomes according to the GRADE framework, driven by the impossibility of blinding clinicians to the respiratory support device, which introduces a high risk of performance and detection bias [14]. Second, the clinical definition of treatment failure was heterogeneous across the included studies, ranging from objective physiological parameters (e.g., escalating pCO₂) to subjective clinical assessments (e.g., increased work of breathing), complicating the direct comparability of this outcome. Finally, data concerning localized adverse events were sparse, limiting the statistical power required to draw definitive conclusions regarding the relative safety of HFNC.

## Conclusions

HFNC and NIV showed no statistically significant differences in intubation rates or PICU LOS. While observational data suggest higher initial failure rates with HFNC, RCT data showed no statistically significant difference in treatment failure between the two modalities. Given its ease of use, patient tolerability, and potential for fewer localized adverse events, HFNC may represent a reasonable first-line noninvasive respiratory support modality. Future research should prioritize large multicenter RCTs with standardized, objective criteria for treatment failure to further elucidate optimal step-up algorithms for these vulnerable patients.
